# Carotegenic *Virgibacillus halodenitrificans* from Wadi El-Natrun Salt Lakes: Isolation, Optimization, Characterization and Biological Activities of Carotenoids

**DOI:** 10.3390/biology11101407

**Published:** 2022-09-27

**Authors:** Doaa Fayez, Asmaa Youssif, Soraya Sabry, Hanan Ghozlan, Marwa Eltarahony

**Affiliations:** 1 Botany and Microbiology Department, Faculty of Science, Alexandria University, Alexandria 21321, Egypt; 2 Environmental Biotechnology Department, Genetic Engineering and Biotechnology Research Institute (GEBRI), City of Scientific Research and Technological Applications (SRTA-City), Alexandria 21934, Egypt

**Keywords:** carotenoids, pigments, halophiles, antimicrobial, COVID-19, MDR, antibiofilm, soda lakes, central composite design

## Abstract

**Simple Summary:**

As one of the two cases of soda lakes in the world, Wadi El-Natrun salt lakes are the intended isolation source in this study. They encompass a characteristic microbial niche of symbionts producing unique bioactive agents, among them carotenoids. Herein, halophilic carotenoid-producing microbes were isolated and purified. Based on the carotenoids content, the potent isolate, namely *Virgibacillus halodenitrificans,* was molecularly identified, and characterized physiologically, biochemically and morphologically as well. Its carotenoids content was optimized using statistical methods. Under optimized conditions, the maximum productivity was obtained and the extracted carotenoids were identified by UV-Vis analysis, FTIR, Raman spectroscopy, TLC and LC–MS, which confirmed such halophilic carotenoids composed of β-carotene, lutein and β-Apo-8′-carotenal mixture. Meanwhile, the carotenoids were utilized as a powerful antibacterial and antifungal agent. Notably, they exerted higher antibiofilm potentiality at a concentration of 20 μg/mL. Finally, halophilic carotenoids are competing as new biocidal agents, in particular with their biocompatibility and efficiency.

**Abstract:**

Carotenoids, as phytonutrient pigments, are signified by their unique beneficial features that serve human health and the surrounding ecosystem. Haloalkaliphiles from soda lakes produce different natural bioactive molecules; however, their ability to produce carotenoids has been limited. Therefore, this study focused on the screening and isolation of carotenoid-producing haloalkaliphilic microbes. Out of 10 isolates, a powerful carotigenic bacterium was isolated, characterized phenotypically and identified on the molecular level as *Virgibacillus halodenitrificans*. By employing statistical approaches like Plackett–Burman design and central composite design, the influence of significant nutritional variables on carotenoids production was screened and optimized. Predictive modeling manifested that carotenoid yield was 36.42 mg/mL, a 2.12-fold enhancement compared to the basal conditions through inoculating 1.8% of bacterial biomass on optimized medium containing yeast extract (2 g/mL), peptone (10 g/mL) and NaCl (233.6 g/mL). The carotenoids content was confirmed by UV-Vis spectrum which detected a characteristic unique peak with left and right shoulders at 461 nm, 490 and 522 nm. However, FTIR and Raman spectroscopy showed the presence of several functional groups. Meanwhile, LC–MS confirmed that the examined carotenoids were composed of β-carotene, lutein and β-Apo-8′-carotenal mixture. As a bioactive agent, the carotenoids of *V. halodenitrificans* DASH showed characteristic antagonistic potency against a wide spectrum of Gram-positive and Gram-negative pathogens. Interestingly, a potent antifungal capacity was observed against *Candida albicans*, reflecting promising mycocidal efficacy against COVID-19 white fungal post-infections. Furthermore, carotenoids (20 μg/mL) inhibited the biofilm formation of *P. aeruginosa* and *S. aureus* by 54.01 ± 3.97% and 80.082 ± 0.895%, respectively. Our results proposed that haloalkaliphiles of Wadi El-Natrun lakes are promising sources of carotenoids that exhibited efficiency as safe, biocompatible and natural bioactive agents for environmental, medical and industrial applications.

## 1. Introduction

Microorganisms are a substantial source of diverse natural products that are candidates for drug development, food, feed additives, and several pharmaceutical and industrial products. Carotenoids are grouped among such brilliant microbial bioproducts, and are signified by their yellow-orange to red-colored pigments and produced by both terrestrial and marine creatures. Their intrinsic role is in photo-protection of photosynthetic organisms from detrimental extra light dosage during photosynthesis [1]. As bioactive secondary metabolites, carotenoids exhibit a vast array of applications as colorants, feed additives, antioxidants, anti-tumor and heart disease prevention agents and precursors of vitamin A. Hence, they are widely applied in the food, medical, pharmaceutical, and cosmetic industries as dyes and functional ingredients [2,3,4,5].

Generally, such types of lipid-soluble pigments are widely synthesized using chemical approaches. However, consumer preference tends towards utilizing natural products that overcome high costs without generating by-products that cause damaging effects to the environment. Via microbial production of natural carotenoids, as a biological alternative source, considerable amounts could be obtained in large scale processes through fermentation [3]. The production of natural colorants through fermentation has a number of advantages, such as cheaper production, higher yields, possibly easier extraction, less batch-to-batch variation and no seasonal variation. The production is flexible and can easily be controlled. Furthermore, the collection of microbial organisms is sustainable and has no negative impact on the environment [4].

Interestingly, hypersaline environments, as extreme habitats, are considered to have a repertory of diverse microbial populations that possess versatile physiological properties and exhibit brilliant functional potentialities. Plenty of bioactive compounds extracted from hypersaline dwelling organisms, including carotenoids, have been reported in prominent biotechnological applications [5,6]. As stated by Waditee-Sirisattha et al. [7], halophilic carotenoids have been utilized as antioxidant, antitumor, immune boosting and protecting-against-premature-ageing agents; moreover, they display outstanding properties for enhancing in vitro antibody production. Furthermore, other studies employed halophilic carotenoids as antimicrobial agents to conquer nosocomial infections caused by pathogens, in particular multidrug resistant microbes (MDR) [8,9].

It is worth looking at the Wadi El-Natrun salt lakes which are one of two cases of soda lakes in the world for natural salt production (natron) besides the Florida saline lakes in the USA. They are located in northern Egypt (23 m below sea level), with coordinates that lie between latitudes 30°17′ to 30°19′ N, and longitudes 30°10′ to 30°25′ E. The geological, hydrogeological, and mineralogical studies revealed that Natron (Na_2_CO_3_·10H_2_O) or Trona (Na_2_CO_3_·NaHCO_3_·2H_2_O), sodium sulfate (Na_2_SO_4_, thenardite) and Halite (NaCl) are the main constituents of Wadi El-Natrun’s saline deposits [10]. Therefore, the microbial diversity within such a characteristic niche could offer prominent symbionts producing unique bioactive agents. Nevertheless, in such unusual niches the exploitation of halophilic microbes as a reservoir of novel bioactive molecules, including carotenoids, is accounted as being in its early stages [1]. 

Thus, the geomorphological features of Wadi El-Natrun’s saline lakes are deemed as being a privileged source for the screening and isolation of halophilic carotenoid-producing microbes that could be harnessed in defeating different forms of microbial pathogens. The present study was taken up with an objective to isolate, characterize and optimize pigments extracted from halophilic bacteria isolated from Wadi El-Natrun’s saline lakes. Thereafter, the antimicrobial performance of the extracted pigments was examined against different bacterial types, either in free or biofilm state.

## 2. Materials and Methods

### 2.1. Samples Collection, Screening and Isolation of Halophilic Carotenoid-Producing Bacteria

Different sediments and water samples were collected from the Wadi El-Natrun salt lakes, which were characterized by high salinity that ranged from 1.5 to 5 M and pH recorded 11. The samples were kept in sterile containers, transferred to the lab within 24 h and stored at 4 °C. The samples were homogeneously mixed and 10 g from each sediment/salt rocks and 10 mL from water samples were suspended in 90 mL sterile distilled water and shaken at 120 rpm for 30 min [11]. Sample extracts were diluted and cultivated within 24 h of sampling on IRAM plates, with the following ingredients (g/L): Peptone, 5 g; NaCl, 116.8 g; MgSO_4_·7H_2_O, 20 g; yeast extract, 4 g; agar, 17 g; KCl, 5 g; and CaCl_2_·6H_2_O, 0.2 g. The pH was 9.0 ± 0.2. The plates were incubated at 30 °C for 7 days. Only orange colonies were selected, then picked up and recultivated on the same medium to obtain pure cultures for further steps.

### 2.2. Molecular Identification of the Selected Halophilic Carotenoid-Producing Isolate

Using the ZR Bacterial DNA MiniPrep™ kit (Zymo Research, Irvine, CA, USA), a bacterial genomic DNA was isolated following the identical protocol. The 16S rDNA gene was amplified using 27F/1492R universal primer to amplify the gene with approximately 1500 bp. The purified PCR product was sequenced and checked in the National Center for Biotechnology Information (NCBI) GenBank database using the BLASTn program for obtaining its phylogenetic affiliation [12].

### 2.3. Morphological and Physiological Characterization of Selected Isolate

The micro-morphological properties of the selected halophilic isolate were characterized by scanning electron microscope. The bacterial cells were prepared, fixed in glutaraldehyde (3%, *v/v*), washed and post-fixed in 1.5% osmium tetroxide for 2 h. The samples were washed, dehydrated by ethanol (40–100%), coated with gold and examined at 15–20 kV by SEM-JEOL JEM-1230-Japan. Regarding its physiological traits, the effect of pH (5–11), NaCl concentration (0 M, 1 M, 2 M, 3 M, and 5 M) and temperature (10 °C, 20 °C, 30 °C and 40 °C) were evaluated on the growth of the halophilic carotenoid-producing strain. The flasks were incubated at 30 °C for 8 days on a rotary shaker (150 rpm). Growth was determined by measuring optical density at OD600 nm with JENWAY6305 spectrophotometer. Each test was implemented in triplicate and the results were displayed by the replicates means ± standard error of the mean (SEM). Additionally, the capability of the selected strain to produce some extracellular hydrolytic enzymes was assessed using the agar plate technique [13]. LB agar plates were supplemented with 10% of skim milk, cellulose powder, starch, or vegetable oil to test for caseinase, cellulase, amylase and lipase, respectively. 

### 2.4. Pigment Extraction 

The selected halophilic producing pigment strain was inoculated on IRAM broth medium and incubated as mentioned previously. The bacterial pellets were obtained by centrifugation at 12,000 rpm for 15 min at 4 °C and subjected to extraction using cold acetone-methanol (7:3 *v*/*v*). The extraction step was repeated successively until reaching pale yellow residues. The pigment extract was evaporated at 45 °C overnight. The dried pigment was weighed, resuspended in methanol and subjected to further analysis [6].

### 2.5. Optimization of Pigment Production by Statistical Design of Experiments (DOE)

DOE is an efficient tool to design, manipulate, analyze and interpret the influence of multiple input parameters (independent variables) on a particular output (response). Through such an approach, optimization of experimental factors and subsequent prospective scaling up would be performed. In this study, DOE was conducted through two successive steps, Plackett–Burman statistical design followed by central composite design (CCD). 

#### 2.5.1. Screening of Significant Independent Variables Influence the Pigments Production by Plackett–Burman Design (PBD)

PBD is a fraction of a two-level factorial design that is devoted to screening and identification of the input factors (nutritional and incubation experimental conditions) depending on their main effect on maximizing pigment production. It is designed to examine ‘n − 1’ variables with at least ‘n’ trials. Each examined variable is represented at two levels, high (+) and low (−) [14]. According to PBD, the number of (+) is equal to (N + 1)/2 and the number of (−) is equal to (N − 1)/2 in a row. A column should contain an equal number of (+) and (−) signs. The main effect was calculated as the difference between the average of measurements assessed at the high setting (+1) and the average of measurements observed at low setting (−1) of each factor. In the current study, a total of 8 (n) variables with two-level concentrations were studied in twelve experimental matrices as indicated in Table 1. Plackett–Burman experimental design is based on the first order model (Equation (1)):

Y = βo + ΣβiΧi(1)

where Y is the response or dependent variable (pigment production); βo is the model intercept and βi is the linear coefficient; and Xi is the level of the independent variable. The significance of each independent variable depending on their nature (i.e., positive or negative effect on the response) was elucidated by main effect that was concluded from the statistical analysis.

#### 2.5.2. Central Composite Design (CCD) Method

At this stage, the interaction among significant variables and also their optimal levels were analyzed and determined [15]. Herein, four significant factors that were deduced from PBD that significantly influenced pigment weight were examined at five experimental levels: –α, –1, 0, +1, and +α in a 31-trial matrix (Table 3). The concentrations of the screened variable at each level were also illustrated in Table 3. For statistical calculation, the relationship between the coded and actual values is described by Equation (2):

Xi = Ui − Ui0/ΔUi(2)

where Xi is the coded value of the ith variable; Ui is the actual value of the ith variable; Ui0 is the actual value of the ith variable at the center point; and ΔUi is the step change of variable. The second order polynomial model that describes the relationship between response (pigment weight/mg) (Y) vs. the significant independent variables is represented in Equation (3):

Y = β0 + β1X1 + β2X2 + β3X3 + β11X11+ β22X22 + β33X33+ β12X1X2 + β13X1X3 + β23X2X3(3)

where Y is the predicted response; X1, X2, X3 are input variables which influence the response variable Y; β0 is intercept; β1, β2 and β3 are linear coefficients; β11, β22 and β33 are squared or quadratic coefficients; and β12, β13, and β23 are interaction coefficients.

#### 2.5.3. Statistical Analysis

The statistical software Minitab 14.0 (Minitab Inc., Pennsylvania, State College, PA, USA) was used to perform the experimental matrices and statistical analysis of PDB and CCD. The regression analysis of the obtained results was employed to calculate the analysis of variance (ANOVA). To determine the relationship between the response and the different levels of each tested variable, three-dimensional surface plots and two-dimensional contour plots were constructed using the same software. Finally, the optimizer tool was utilized to predict the optimum concentrations of experimental factors for maximizing response [16].

#### 2.5.4. Validation of Experimental Model

The statistical model was evaluated by determining pigment production under conditions predicted by the model and by comparison of the values to those obtained from the basal media.

### 2.6. Characterization of Extracted Pigment

#### 2.6.1. UV-Vis Spectra Absorption

The characterization of extracted pigments was initiated by scanning the absorbance in the wavelength region of 200–800 nm to find out the maximum absorption spectra (methanol was used as a blank) [17].

#### 2.6.2. Raman Spectroscopy

The influence of the excitation wavelength on the pigment spectrum was determined by using Raman Senterra instrument. The sample was excited at room temperature and atmospheric pressure by exposing them to a laser beam of 785 nm with a wide range 400–4000 cm^−1^ for 1 s at 50 mW power. The light scattered from the sample was gathered by the microscope optics, which passed through holographic filters, pinhole, and monochromatic and was detected by a charge-coupled device [6].

#### 2.6.3. Fourier-Transform Infrared Spectroscopy (FTIR) Analysis

The functional groups of pigment molecule were identified using Schimadzu FT-IR Affinity-1 Spectrometer. A mixture of about 1 mg of the pigment and 300 mg of pure dry potassium bromide (KBr) were pressed into discs. The measurements were carried out at infrared spectra between 400–4000 cm^−1^ at a resolution of 4 cm^−1^.

#### 2.6.4. Thin-Layer Chromatography

In order to identify carotenoid pigments in the extract, thin-layer chromatography (TLC) was employed. The methanol extract was placed on a TLC silica gel GF254 plate as Stationary phase (Merck, Darmstadt, Germany) and developed in petroleum ether: acetone (80:20) as a mobile phase. After development, the individual spots were identified by visibility and by UV screening. Retention time was calculated [18].

#### 2.6.5. LC–MS

The extracted pigment was analyzed by electrospray ionization mass spectrometry (ESI-MS) using Agilent Technologies 6420 Quad LC/MS system (Germany). In an analytical procedure proposed for the separation of analytes from the mixture of carotenoids, the mobile phase was composed by acetonitrile: methanol: ethyl acetate (*v/v*) in a gradient, from 95:5:0 to 60:20:20 in 20 min, the latter proportion being maintained until the end of the run. Flow rate was set at 0.5 mL min^−1^ and injection volume was 10 μL. Acetonitrile contained 0.05% of triethylamine to improve carotenoid recovery from the chromatographic column. UV-Vis was used, with detection being at the wavelengths of maximum absorption λ = 490 nm. 

### 2.7. Stability of Pigment

The stability of crude pigment extract was determined in terms of its absorbance at λmax. The effect of various conditions of pH (5, 7, 9) on stability was examined at room temperature. The pH showing maximum stability was selected to investigate effect of temperature (−18, 5, 30, 50 °C). The effect of different concentrations of salt (1 M, 2 M, 3 M) was also assessed. Moreover, pigment stability under dark and light conditions was detected [19]. Each trial was carried out in triplicate and the results were expressed as means ± SEM.

### 2.8. Biological Activity of Halophilic Carotenoids 

#### 2.8.1. Antimicrobial Activity

The antagonistic potency of extracted pigment was examined against different pathogens (*Staphylococcus aureus* ATCC 25923, *Escherichia coli* ATCC 25922, *Pseudomonas aeruginosa* ATCC 27853, *Enterococcus faecalis* ATCC 29212 and *Candida albicans* ATCC 10231). About 100 μL of standardized inoculum of each test organism (10^7^ CFU/mL) was inoculated on petri dishes containing nutrient agar medium (for bacteria) and Sabauraud’s agar (for yeast) under aseptic conditions. A standard corkborer of 6 mm in diameter was used to make uniform punctures, into which 60 μL of 20 μg/mL of pigment extract was added. Methanol was used as negative control. The plates were then incubated at 37 °C for 24 h. After incubation, the zone of inhibition was measured. Each experiment was conducted in triplicate and the results were displayed by the replicates means ± SEM [20].

#### 2.8.2. Antibiofilm Potency

The efficacy of pigment extract to inhibit biofilm formation was evaluated using microtiter plate assay. Bacterial suspensions of (*Staphylococcus aureus* ATCC 6538 and *Pseudomonas aeruginosa* ATCC 9027) were prepared in tryptic soy broth (TSB) and 200 μL of bacterial suspension was inoculated into a 96-well flat-bottomed sterile microtiter plate. Untreated wells containing only cell suspension (biofilm formed), and also uninoculated wells containing sterile TSB, were considered as negative control wells. Additionally, wells containing bacterial suspensions were treated with methanol to normalize the readings of carotenoids-treated wells; the inhibition effect of methanol was subtracted from overall inhibitory effect of both carotenoids’ doses. Cells were separately exposed to different concentrations of pigment extract (10 and 20 μg/mL) and standard antibiotic chloramphenicol (10 and 20 μg/mL) in triplicates. The inoculated plate was incubated at 24 h at 37 °C. The formed biofilms in the microplate were stained with 150 μL of crystal violet for 15 min; after planktonic cells were discarded, it was washed twice with phosphate-buffered saline PBS (pH 7.2). The stained wells were resolubilized by 150 μL of 95% ethanol and then the microplate was measured spectrophotometrically at 570 nm by a microplate reader (Tecan Infinite M200, Switzerland) [20]. The inhibition percentage of biofilm was calculated as described by the following equation:

Inhibition percentage of biofilm = [(A0 − At/A0) × 100](4)

where A0 denotes the absorbance of the positive control and At the absorbance of the treated well.

## 3. Results

### 3.1. Screening, Isolation and Molecular Identification of Halophilic Carotenoid-Producing Bacteria

**Figure 1:**
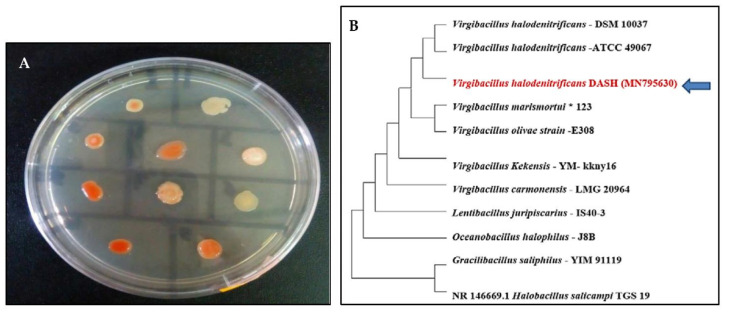
IRAM plate showing bacterial isolates screened from Wadi El-Natrun’s Salt Lakes with different pigment production capabilities (**A**) and the phylogenetic tree showing the position of the selected halophilic bacterial isolate *Virgibacillus halodenitrificans* DASH- MN795630 (**B**).

The current study focused on the pigment production from halophilic bacterial isolates screened from one of the most characteristic hypersaline ecosystems in Egypt. Only 10 isolates were obtained on IRAM agar plates (Argentine Institute of Standardization and Certification), based on their capability to produce pigments. As observed in Figure 1, some of the colonies obtained showed orange color with different degrees, while others had creamy and pink color (Figure 1A). Accordingly, the isolate designed as DASH was selected, which was also distinguished by additional outstanding biochemical and physiological potentialities relative to the others. Thereafter, about 1434 bps of 16S-rDNA gene was sequenced from the isolate DASH followed by homology search using Blastn analysis. The multiple sequence alignment showed 99% identity with the sequences of *Virgibacillus halodentificans*. Subsequently, the isolate was identified as *Virgibacillus halodenitrificans* DASH and its sequence was deposited in the GenBank under the accession number of MN795630. Additionally, the phylogenetic tree was constructed based on 16S rDNA gene sequences of *Virgibacillus halodenitrificans* DASH and members of closely related species of genus *Virgibacillus* by utilizing the neighbor-joining method. Figure 1B depicts the proximate vicinity of our strain with *Virgibacillus halodenitrificans* DSM-10037 and *Virgibacillus halodenitrificans* ATCC-49067.

### 3.2. Phenotypic Characterization

**Figure 2:**
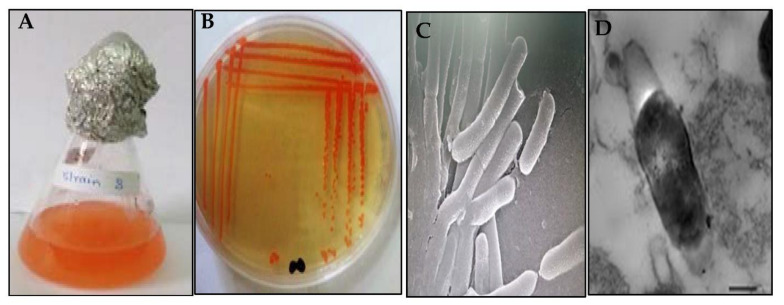
Cultural and morphological characteristics of *V. halodenitrificans* DASH. (**A**) Pigmented IRAM broth after one week incubation, (**B**) Small round orange colonies on IRAM agar, (**C**) Cell morphology by SEM and (**D**) Cell morphology by TEM showing central ellipsoidal endospore.

The cultural traits of *V. halodenitrificans* DASH, after 7 days of incubation at 37 °C on IRAM plates, displayed small round, smooth, shining, and moist colonies with neat edges and deep orange pigmentation. Generally, its colonies were easy to pick up (Figure 2A,B). Regarding the morphological properties, its cells are long-rods and Gram-positive with ellipsoidal central endospore as observed by scanning electron microscopy (SEM) and transmission electron microscopy (TEM) after 15 days of incubation (Figure 2C,D).

### 3.3. Physiological Characterization

**Figure 3:**
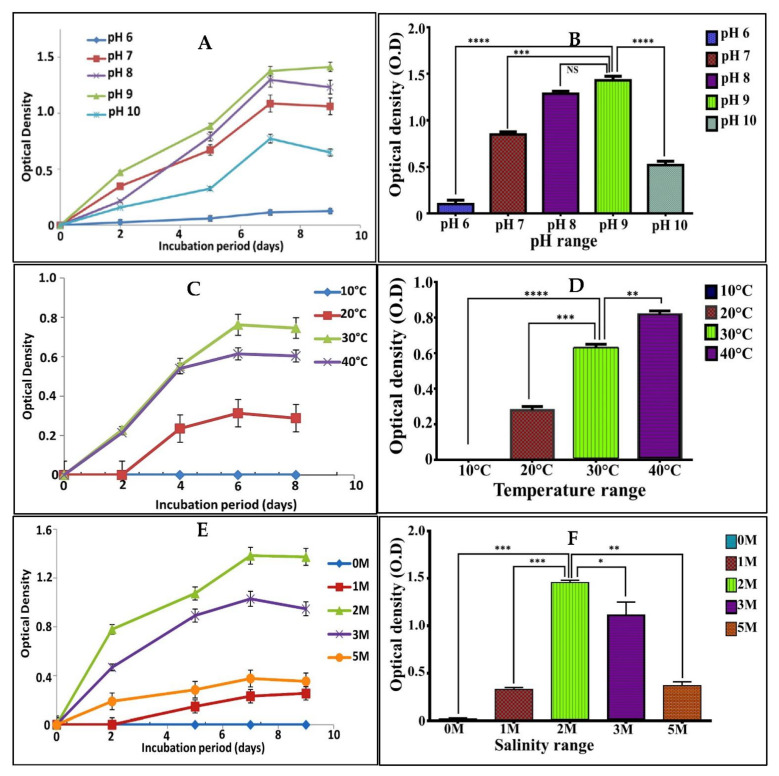
Physiological characteristics of *V. halodenitrificans* DASH during 9 days of incubation. (**A**,**B**) pH, (**C**,**D**) temperature and (**E**,**F**) different concentrations of NaCl. All values were expressed as mean ±SEM. This comparison considers significantly different at * *p* < 0.05, ** *p* < 0.005, *** *p* < 0.0005 and **** *p* < 0.0001, and NS (non-significant) as indicated by multiple comparisons Tukey post-hoc analysis of variance (ANOVA).

The variation in pH, temperature and NaCl concentrations revealed that *V. halodenitrificans* DASH was alkaliphilic; it exhibited significant good growth at 9 (*p* < 0.0001), with optical density reaching 1.4 ± 0.75, while the growth decreased gradually at both higher and lower values of pH. Moreover, the optimum temperature for its growth was recorded at 30 °C (O.D = 0.75 ± 0.11) with remarkably significant lowering at higher and lower temperature. Furthermore, as revealed by one-way ANOVA, no obvious growth recorded in media lacked NaCl; whereas, optimum and significant growth was recorded in the presence of 2M of NaCl (O.D = 1.45 ± 0.91) and declined in all other examined concentrations (Figure 3). Concerning its enzymatic potentiality, *V. halodenitrificans* DASH exhibited positive response to protease, cellulase, lipase and negative results for amylase. The presence of a clear zone formed around the growth was taken as evidence of enzymatic activities [21,22,23].

### 3.4. Optimization of Pigment Production Using Experimental Design

#### 3.4.1. Screening of Significant Independent Variables Influencing Pigment Production by Plackett–Burman Design (PBD)

Design of experiment (DOE) is a collection of statistical and mathematical inferences employed for identifying the optimum conditions for a multivariable system, constructing and exploring an approximate functional interaction between a response variable and a set of design variables techniques [24]. The use of a good, reliable statistical model is essential to develop better strategies for the bioproducts optimization process. In this study, the most significant independent variables for maximum pigment weight were determined through PBD, which displayed extreme disparity ranging from 0.368 mg/mL (trial number 5) to 29.47 mg/mL (trial number 7) (Table 1). Such results differences implied the vital role of the optimization process in elevation of pigment productivity. The analysis of multiple linear regression coefficients of the model was carried out using MINITAB 14 via the Student’s t-test. Supplementary Table S1 illustrated the coefficient of each variable representing the effect extent of this variable on pigment weight and also their *p*-values. In general, *p*-value reveals the significance and consequences of each independent variable within the design; the larger the magnitude of the t-value and smaller the *p*–value (prob > F < 0.05), the greater is the significance and effect of the corresponding coefficient term on the response [25]. Based on the calculated *p*-value, NaCl (*p*-value, 0.002), peptone (*p*-value, 0.014), yeast extract (*p*-value, 0.026) and inoculum size (%) (*p*-value, 0.038) were the most significant media components that affected pigment production, as obviously demonstrated in the probability plot of effects and in the Pareto charts (Supplementary Figure S1A,B).

**Table 1:** Twelve-experiment Plackett–Burman matrix for evaluation of independent factors with high/ low values along with the actual and predicted response (pigment production).

Run Order	KCl	MgSO_4_	Y.E.	Peptone	NaCl	Inoculum Size (%)	pH	Volume/Flask (mL)	Experimental Pigment Weight	Predicted Pigment Weight
	(g/L)								(mg/mL)	
1	2.5	40	8	2.5	233.6	0.5	6	50	13.05	14
2	2.5	10	8	10	233.6	0.5	9	150	21	21.42
3	10	10	8	2.5	29.2	0.5	9	150	0.57	0.157
4	10	40	8	2.5	233.6	5	6	150	7.89	6.94
5	2.5	40	2	2.5	29.2	5	9	150	0.368	0.368
6	2.5	10	2	2.5	29.2	0.5	6	50	8.63	7.68
7	10	40	2	10	233.6	0.5	9	50	29.47	27.3
8	2.5	10	2	10	233.6	5	6	150	22.31	21.89
9	10	10	2	2.5	233.6	5	9	50	14.31	16.42
10	2.5	40	8	10	29.2	5	9	50	2.21	2.94
11	10	10	8	10	29.2	5	6	50	4.78	4.05
12	10	40	2	10	29.2	0.5	6	150	9.52	11.63

Further, normal probability plot (NPP) is the graphical method that can be used to validate models and also characterize the nature of residuals of the models (Supplementary Figure S1C). The residuals are the difference between the actual or observed responses and the responses predicted by the theoretical model (Table 1). The adequate model with its distribution describes as normal when the points on the normal probability plots of the residuals approximately lie on a straight line (Supplementary Figure S1C) [26,27].

The standard analysis of variance (ANOVA) of the Plackett-Burman design manifested that the model was highly significant as was evident from the low probability value [*p*-value = 0.017] (Table 2). In addition, the overall performance of the model was examined through assessing the coefficient of determination (R^2^) and the adjusted-R^2^ (adj-R^2^) value, which should be in reasonable agreement with the R^2^ value (less than 2%) [20,28]. As referred to by Abu-Elreesh et al., 2019 [15], the stronger model with better prediction of response takes place with R^2^ value closer to 1. Herein, the model R^2^ and adj-R^2^ values recorded for pigment production were 0.9807 and 0.9294, respectively. Such results indicate that 98.07% of the variability of the data can be explained by the model, and there is only a 1.93% chance that it could be due to noise. The first order model for pigment weight obtained by ANOVA was fitted to the results obtained from the 12 experimental trials, which elucidated the pigment weight as a function of eight studied parameters (Equation (5)).

Pigment weight (g) = 0.212 − 0.0016 KCL − 0.0144 MgSO_4_ − 0.0556 Yeast Extract + 0.0704 Peptone + 0.130 NaCl − 0.0481 Inoculum Size + 0.0028 PH − 0.0171 Volume/flask (5)

For the second step of optimization (central composite design CCD), all parameters with a positive influence on pigment weight were kept constant at their high level, and those factors negatively affecting it were maintained at their low level.

#### 3.4.2. Central Composite Design (CCD) for Optimization of Pigment Production

At this stage, the interactions among the significant parameters and the accurate estimation of their optimum levels were studied as well. On the basis of PBD results, NaCl, peptone, yeast extract and inoculum size were selected for further optimization by a five-level CCD in a 31-run matrix consisting of 16 factorial (cubic points), 8 axial or star points (points having an axial distance to the center of (α = ± 2)), and 7 replicates of center points, as the risk of missing non-linear relationships in the middle of the intervals has to be minimized and the repetition allows determining confidence intervals [14,29]. The design matrix, the concentrations of examined parameters at their coded and actual levels, along with the experimental and predicted responses and the studentized residuals, are displayed in Table 3. As noticed, pigment productivity varied considerably among the experimental runs, exhibiting maximum response with 23.1 mg/mL at trial 29 (central point) and minimum response with 0.21 mg/mL at trial 18 (axial point) (Table 3). 

#### 3.4.3. Multiple Regression Analysis and ANOVA

The CCD data were analyzed using multiple regression analysis and ANOVA to attain the significance of each of the individual model coefficients, the significance of the regression model and the lack-of fit (Supplementary Table S2). The determination coefficient R^2^ and adj-R^2^ were applied to evaluate the goodness of fit of the model. In our investigation, R^2^ recorded 0.938, reflecting that 93.8% of variation in pigment weight was explained by the tested variables and only 6.2% of the variations are not explained by these factors. Moreover, adj-R^2^ recorded 0.889, reflecting a reasonable agreement and good adjustment with R^2^ value. However, the lack-of-fit test, which describes the variation in the data around the fitted model, was assessed at 0.128 as inferred by ANOVA. Generally, insignificant lack-of-fit denotes a good model [30]. Notably, the ANOVA result, which is summarized in Supplementary Table S2, showed that the model was highly appropriate and adequate as concluded from the low probability value (*p*-value, 0.000). 

Moreover, the residuals generally fall on a straight line with no reasonable outliers, implying that the errors are distributed normally, which could be concluded from normal probability plots of the residuals (Supplementary Figure S2). Subsequently, the graph displayed that the predicted values were found to be statistically similar to the actual measured values. In addition, the studentized residuals, which is the residual divided by an estimate of its standard deviation, fall in an acceptable range (less than ±2) [30] (Table 3). Therefore, all these results indicated that the model is significant, adequate and well-fitted to the experimental data.

Furthermore, multiple regression analysis pointed out the effect of each individual variable, squared and their second-order interactions on pigment production according to their sign (positive or negative), and the statistical significance of their coefficients (*p* < 0.05). The probability values of the coefficients showed that the quadratic effects of the tested variables had a more predominant effect in enhancing pigment productivity than linear and interaction effects (Supplementary Table S2). Additionally, the linear coefficients of both yeast extract and inoculum size indicated their significant role in improving pigment yield where any little changes in their concentrations will alter the pigment weight; consequently, both consider being limiting factors. Furthermore, the interaction effect of all tested variables seemed to be insignificant, with the exception of the interaction between yeast extract and inoculum size, and between peptone and NaCl. Moreover, the interaction effect between the pairs yeast extract and inoculum size, yeast extract and NaCl and inoculum size and NaCl, could be described as antagonistic, and their negative coefficient showed a higher amount of pigment yielded upon increasing the concentration of one factor and decreasing the concentration of the other one. Finally, the pigment weight as a response can be expressed in the terms of a second order polynomial equation (Equation (6)).

Pigment weight (g) = 0.401 + 0.027 Yeast Extract − 0.006 Peptone + 0.004 NaCl + 0.043 Inoculum size − 0.065 (Yeast Extract)^2^ − 0.030 (Peptone)^2^ − 0.099 (NaCl)^2^ − 0.023 (Inoculum size)^2^ − 0.01 Yeast Extract* Peptone − 0.0003 Yeast Extract* NaCl − 0.032 Yeast Extract* Inoculum size + 0.048 Peptone* NaCl − 0.005 Peptone* Inoculum size − 0.006 NaCl* Inoculum size (6)

#### 3.4.4. Graphical Interpretation of the Response Surface Model

**Figure 4:**
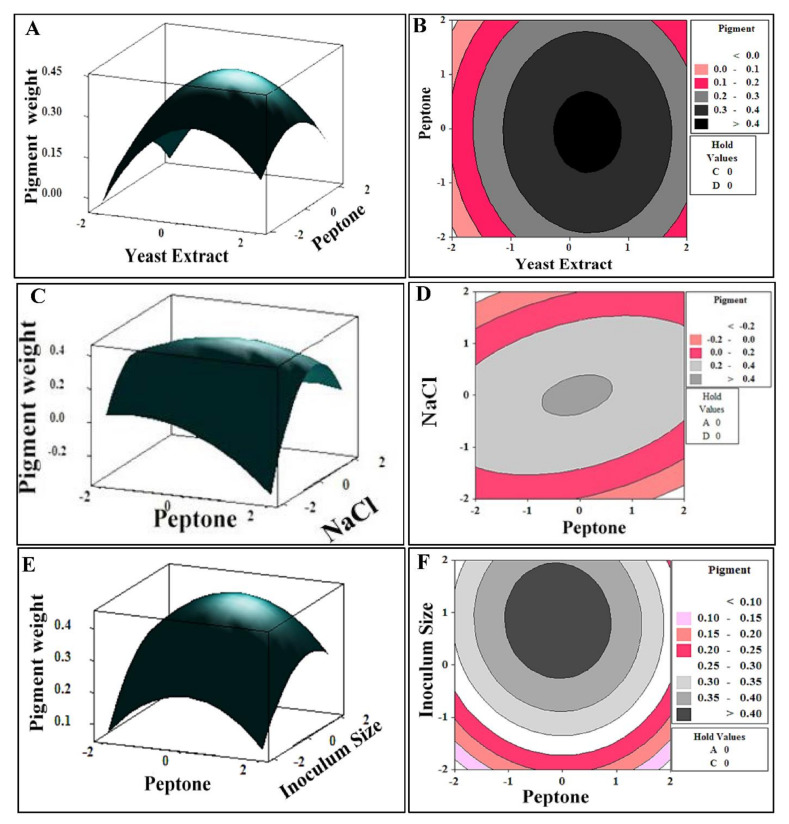
Three- dimensional surface plots (**left** panels) and two-dimensional contour plots (**right** panels) showing the interactive effects of independent significant variables on pigment productivity.

The graphical illustrations of the model equation which explained the relationship between different levels of pair-wise combination of the studied variables and pigment weight were generated by plotting the three-dimensional response surface plot (3D) and two-dimensional contour plots (2D) (Figure 4) [31]. The empirical functional relationship is expressed as the pigment weight (response) on Z-axis against coded levels of two independent factors on horizontal axes, while the other explanatory factors are kept at their center point (zero levels). Figure 4A,B depict a 3D-surface plot and a 2D-contour plot as a function of yeast extract and peptone on pigment weight at constant values of NaCl and inoculum size at their zero levels. It showed that high pigment production was obtained at the middle level of both factors. Moreover, the circular shape contour plot revealed insignificant interaction between the two factors. Generally, the shape of the contour plot indicates the extent and nature of the interactions between the examined variables. Elliptical and saddle-shaped contour plots elucidate a significant interaction between variables, whereas a circular contour plot highlights an insignificant interaction [14,15].

As a consequence, the correlation between peptone and NaCl was deemed as being significant (Figure 4C,D). Maximum pigment weight could be achieved by increasing the concentration of both variables simultaneously, implying the synergetic interaction. On the other hand, high inoculum size with low concentration of peptone, and vice versa, would improve pigment productivity as observed in Figure 4E. Insignificant antagonistic effect of the interaction between the two factors and pigment yield was deduced from a circular shape 2-D plot (Figure 4F). The reduced regression model was solved for predicting the maximum pigment production via the Response Optimizer tool in MINITAB 14.0. Minitab’s Response Optimizer calculates individual desirability by a desirability function. Individual and composite desirability assess how well a combination of variables satisfies the goals defined for the responses [32]. Desirability has a range of zero to one. One represents the ideal case and zero indicates that the response is outside its acceptable limits. Thus, the predicted optimal levels of the process variables were as follows (g/L): yeast extract, 2; peptone, 10; NaCl, 233.6; and inoculum size, 1.8%. The results of the response optimizer at optimum condition for maximum goal were shown in Supplementary Figure S3.

#### 3.4.5. Experimental Verification of Model

**Figure 5:**
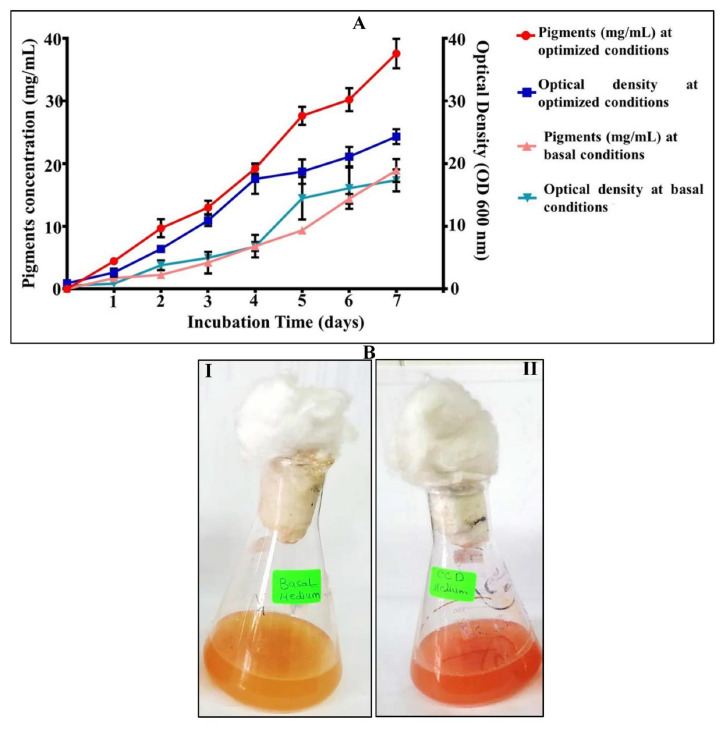
Verification experiment of halophilic carotenoids produced by *V. halodenitrificans DASH*. (**A**) Comparative analysis of carotenoids’ productivity and optical density under optimized and basal medium as a function of incubation time (7 days) with time interval (24 h). The average of three replica were carried out for each one. (**B**) Photographic image representing verification step. I-under basal unoptimized conditions, II-under predicted optimum conditions.

In order to validate the obtained statistical model and ensure its accuracy, an experiment was performed in triplicate using the predicted optimized condition in parallel with basal media before optimization process. The optimization strategy led to a 2.12-fold enhancement of pigment yield from 17.15 ± 2.84 mg/mL (basal) to 36.42 ± 3.41 mg/mL (optimized) (Figure 5A,B).

### 3.5. Stability of the Pigment

**Figure 6:**
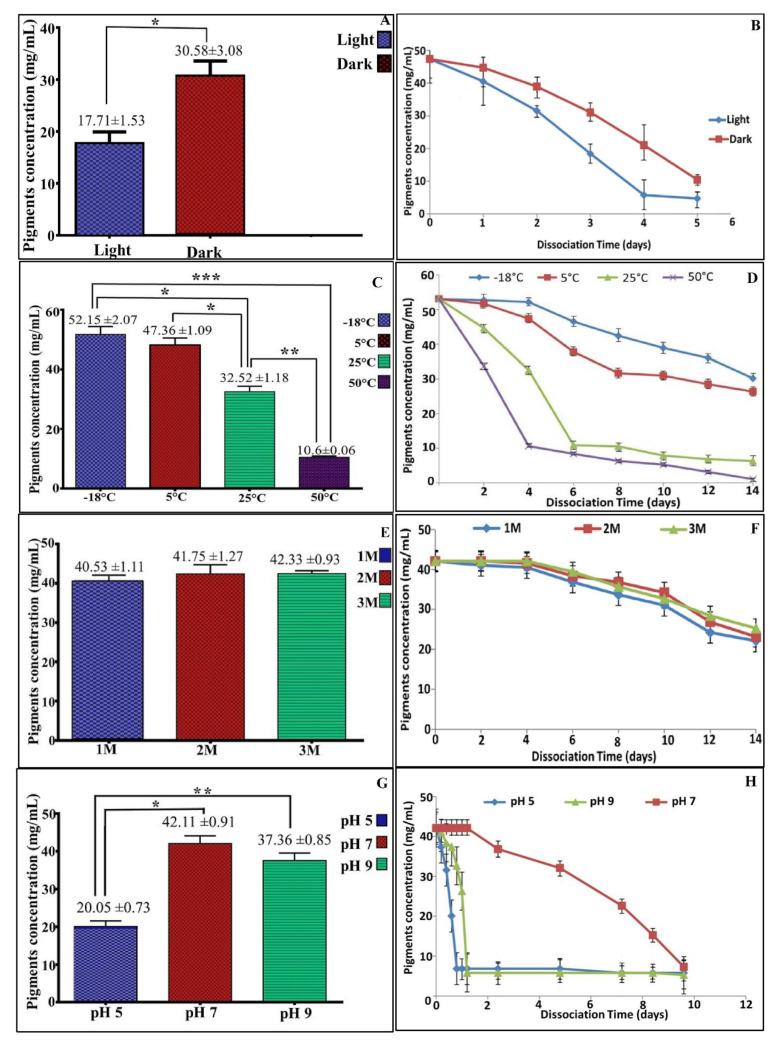
Stability of pigments under different conditions. (**A**,**B**) Effect of light and dark, (**C**,**D**) Effect of different temperatures, (**E**,**F**) Effect of different NaCl concentrations, (**G**,**H**) Effect of pH change. All histograms represent mean ±SEM of the data at 96 h of incubation. This comparison considers significantly different at * *p* < 0.05, ** *p* < 0.005, *** *p* < 0.0005.

Storage stability of any product represents the most significant aspect in terms of industrial applicability. Notably, the poor stability and photosensitivity of natural pigments triggers their market limited. However, the methanolic extract of pigment produced by *V. halodenitrificans DASH* showed less stability (17.71 ± 1.53 μg/mL) when exposed to light during storage, whereas it is significantly more stable (30.58 ± 3.08 μg/mL) for a longer time when stored in dark (Figure 6A,B). Also, we found that the stability of *V. halodenitrificans* DASH pigment is significantly high at the range of 5 °C to −18 °C, recording 52.15 ± 2.07 and 47.36 ± 1.09 μg/mL, respectively; whereas, very poor stability was observed at 25 °C and 50 °C (Figure 6C,D). Notably, the addition of NaCl to the pigment extract led to an increase in the stability of pigment to a certain extent, with insignificant differences between the examined concentrations, as noticed in Figure 6E,F. Furthermore, upon exposure to various pH ranges at room temperature, the results indicated that the pigments exhibited significant stability (*p* < 0.05) and reached approximately 90% at pH 7.0. (Figure 6G,H). The existence of pigment under acidic or basic levels caused dissociation rapidly within a few hours, while neutral pH preserved its stability for a longer time. 

### 3.6. Characterization of Pigment

#### 3.6.1. UV-Vis Spectrophotometer 

**Figure 7:**
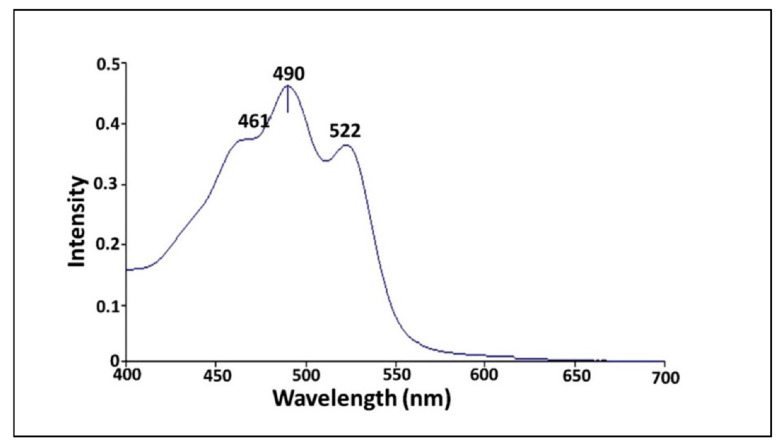
UV-Vis spectroscopy scanning of carotenoid pigments extracted from halophilic *V. halodenitrificans* DASH.

Initially, the extracted pigment from *V. halodenitrificans* DASH was monitored by UV-Vis spectrophotometric in a wavelength scan ranged from 200 to 800 nm. As shown in Figure 7, one major peak and two shoulders on the left and right were observed at 461 nm, 490 and 522 nm, respectively, which is considered characteristic for carotenoid compounds [33].

#### 3.6.2. Raman Spectroscopy and Fourier-Transform Infrared Spectroscopy (FTIR) Analysis

**Figure 8:**
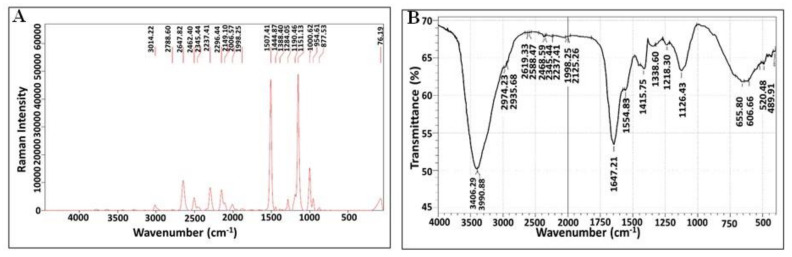
Structural characteristics of carotenoids extracted from halophilic *V. halodenitrificans* DASH. (**A**) Raman spectra of pigment signal and (**B**) FTIR profile.

Both spectroscopic profiles unveiled the presence of various chemical groups in the pigment extract from halophilic *V. halodenitrificans* DASH (Figure 8). As noticed, Raman spectra demonstrated two strong bands in the vicinity of 1151.13 cm^−1^ and 1507.41 cm^−1^ which are characteristic of C-O-H group and CH3 umbrella mode stretching vibrations, respectively (Figure 8A). In addition, other medium peaks centered around 2296.44, 2504.49 and 2788.6 cm^−1^ belong to C-H stretching vibrations. Meanwhile, the peaks at 2149.1, 1190.45, 1000.62 and 3014.22 cm^−1^ are characteristic of C=C, C-CH, C-O stretching vibration and –OH group, respectively [34]. On the other hand, the FT-IR pattern indicated the presence of peaks at 520.78 cm^−1^ and 666.8 cm^−1^, which correspond to C-Br and C-Cl stretching frequency, respectively (Figure 8B). A vibration band at 1126.43 cm^−1^ could be attributed to aliphatic C-O. The wavenumbers of 1319.3 cm^−1^ and 1338.9 cm^−1^ referred to bending –CH3, the band at 1415.75 cm^−1^ is assigned to bending -CH2-, while the band at 1564.6 cm^−1^ could be attributed to aliphatic cycle. The strong peak at 1647.21 cm^−1^ is assigned to the C=O; however, absorbance peaks at wavenumbers 2936.6 cm^−1^ and 2974.2 cm^−1^ refer to stretching aldehyde C-H, while the strong peak at 3406.29 cm^−1^ assigned to phenolic OH [35].

#### 3.6.3. Thin-Layer Chromatography (TLC) and Liquid Chromatography–Mass Spectrometry (LC–MS)

**Figure 9:**
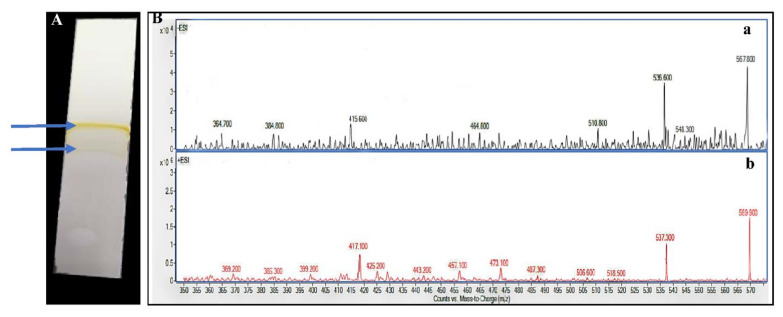
TLC plate (**A**) and LC mass spectral analysis of the pigment extracted from halophilic *V. halodenitrificans* DASH (**B**). For TLC, silica gel GF254 plate (Merck, Darmstadt, Germany); mobile phase: petroleum ether: acetone (80:20). For LC–MS, negative ionization scanning mode (350–600 Da) (a) negative (-ESI) and (b) positive ionization scanning mode (+ESI).

Under UV lamp, two distinct orange and yellow bands were observed with retention time of 0.82 and 0.67, respectively, in addition to a few other invisible bands, as illustrated by TLC analysis (Figure 9A). Meanwhile, LC–MS analysis of the crude methanolic extract of the orange pigment was demonstrated in Figure 9B. It showed a fraction separated at retention time of 0.568 min with a molecular weight of *m*/*z* 567.8, 536.6 and 415.6 according to the negative ionization scanning mode, and *m*/*z* 569.9, 537.3 and 417.1 according to the positive ionization scanning mode. 

### 3.7. Biological Activity of Halophilic Carotenoids

#### 3.7.1. Antimicrobial Activity 

The prevalence of microbial pollution, derived from different fields including water treatment systems, food processing, agricultural products and pharmaceutical/medical industries, aids in the development of effective traditional and commercial antimicrobial agents. However, the abuse and over utilization of antibiotics has caused outbreaks of multidrug resistant microorganisms (MDR) where various and numerous types of microbial species tolerate such antimicrobial mediators via excessive types of barriers and strategies. Consequently, the antimicrobial potency of halophilic carotenoids in the current study was assessed against a vast array of MDR pathogens. The results of well-cut diffusion method pointed to the presence of clear zones of inhibition (ZOI) that varied among the examined pathogens (Supplementary Figure S4A). As referred to by Prasad et al. [36], the effectiveness of any examined antimicrobial agent is evaluated as “good” when the zone of inhibition measures more than 1 mm. Apparently there is no specific trend in the antibacterial activity of halophilic carotenoids in their response against Gram-positive and Gram-negative bacteria, despite the obvious variation in cell wall structure, composition, permeability and polarity. Hence, the resistance order of examined pathogens versus halophilic carotenoids, based on ZOI, could be summarized as *Staphylococcus aureus* (10 ± 0.2 mm) ˃ *Pseudomonas aeruginosa* (12 ± 0.2 mm) ˃ *Enterococcus faecalis* (21 ± 0.21 mm) ˃ *Escherichia coli* (23 ± 0.42 mm). Moreover, a remarkable ZOI of mycostasis was observed against *C. albicans*, which recorded 25 ± 0.41 mm, reflecting potent antifungal activity of the examined carotenoid concentration (20 μg/mL). 

#### 3.7.2. Antibiofilm Potency 

**Figure 10:**
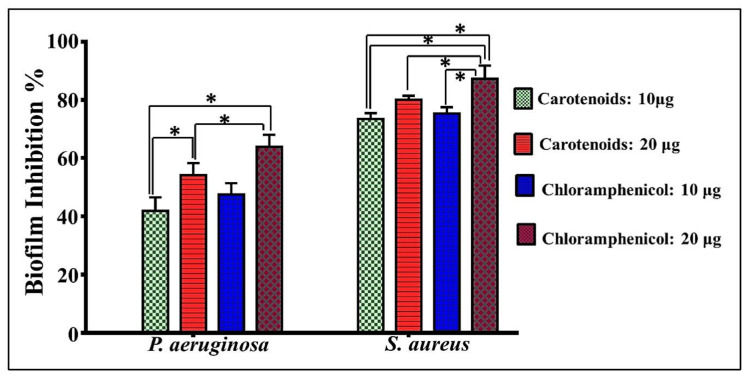
Antibiofilm potency of haloalkaliphilic carotenoids extract and the significant inhibition of both biofilms at both examined concentrations, in comparison to chloramphenicol. The values were mean of three replicas ±SEM. This comparison considers significantly different at * *p* < 0.05.

Biofilms are defined as a collective microbial community enclosing themselves in a self-produced extracellular polymeric matrix (EPS), which is composed of polysaccharides, DNA and proteins. By dint of EPS viscoelasticity, the biofilms attach themselves to a living or inert surface; this is a serious concern, both environmentally and medically, for water purification systems, water pipelines and also for medical tools. In addition, the overall biofilm configuration triggers it to be more resistant to harsh conditions including lethal medication which exceeds the levelsused to treat the planktonic state by three to four fold. In a trial to find efficient antibiofilm agents, the natural microbial products were examined. Hence, in comparison to chloramphenicol, the halophilic carotenoids extracted from *V. halodenitrificans* DASH were studied in eradicating the biofilms formed by *S. aureus* and *P. aeruginosa* as models for Gram-positive and Gram-negative biofilms, respectively. Notably, the biofilm of *S. aureus* showed higher susceptibility than the biofilm of *P. aeruginosa* (Supplementary Figure S4B(a-f)–F)). Further, there was obvious elevation in biofilm inhibition percentage upon increment of the antibiotic and carotenoid dosages. The standard antibiotic showed 47.5 ± 1.92 and 75.4 ± 0.74% prohibition for *P. aeruginosa* and *S. aureus* biofilms at 10 μg/mL, respectively, whereas 20 μg/mL blocked the biofilm growth of *P. aeruginosa* and *S. aureus* significantly by 63.85 ± 1.42 and 87.25 ± 1.29%, respectively. Comparatively, 41.82 ± 3.18% reduction in biofilm formation was recorded in *P. aeruginosa* by using 10 μg/mL of extracted carotenoid. Moreover, significant inhibition was recorded by 54.01 ± 3.97% at 20 μg/mL of alkaliphilic pigments. Likewise, the formation of *S. aureus* biofilm was prohibited significantly by 73.9 ± 1.16% and 80.082 ± 0.895% upon applying 10 and 20 μg/mL of carotenoid, respectively, indicating antibiofilm efficiency in a dose dependent manner (Figure 10).

## 4. Discussion

Soda lakes which originated since the first geological records of the world contain higher concentrations of sodium carbonates. They harbor diverse saline and alkaline niches (haloalkaliphilic) that participate mainly in the cycling of life-essential elements, such as carbon, nitrogen and sulfur. By virtue of such unique geochemical properties, various ecologically and economically important microbial communities appeared. Therefore, abundant new genera and species dwelling in such ecosystems represent valuable models for different molecular, ecology and biotechnology studies [37,38]. In this context, intense investigations have been performed using alkaliphilic microbes and their bioactive molecules which have resulted in several applications to improve human life. Pigments, in particular carotenoids, are categorized among such bioactive molecules [39,40]. Carotenoids were deemed as one of the most important pigments which have been proposed to play an important role in protecting organisms from oxidative damage by such active oxygen species as singlet oxygen (^1^O_2_) produced by a photochemical reaction [41]. Carotenoids were explored for their provitamin A activities, and for their high antioxidant potential, which allows them to act against life-threatening diseases such as cancer, age-related macular degeneration, retarded tooth or bone development and impaired rough scaly skin in humans [42]. 

In recent decades, researchers focused on utilizing microalgae such as *Chlorella* sp., *Muriellopsis* sp., *Scenedesmus almeriensis* and *Coccomyxa acidophila* as rich sources of pigments, which are generated ordinarily in their natural aquatic systems to create a competitive niche [43]. Interestingly, the photosynthetic algal cultivation could be implemented in wastewater, thus evading competition for expensive nutrition and water sources needed for food crops [44]. Notwithstanding this advantage, the produced quantity of carotenoids from wild type strains is deemed insignificant to satisfy industrial and market feasibility, let alone considering the prolonged incubation time, which can reach 30 days, and the restrictive procedures for maintaining optimum growth conditions, biomass recovery and product extraction to guarantee the amounts that would meet market requirements [43]. Thus, the advances in algal photobioreactors (PBRs) have gained great interest in past decades to compensate for these previous drawbacks. However, the low efficacy of the utilized photosynthetically active radiation, namely inhomogeneous light distribution, photoinhibition and photooxidative stress, caused diminishing light-to-biomass conversion efficiency and eventually lower carotenoids yield, which inflates production costs [44]. Hence, a plethora of endeavors were exerted to explore other new microbial taxa that exhibit outstanding economic carotenoids productivity in a more convenient time frame. Such an objective could be fulfilled from new and distinctive isolation sites. Thus, the present study focused on production, optimization and application of pigments produced by haloalkaliphilic microbes screened from the El-Natrun salt lakes as a model of soda lake habitat. 

On IRAM medium, 10 isolates were obtained containing varied pigments content. However, only the strain that contained the higher pigments content was identified and characterized to accomplish the required target. The physiological properties of the strain *Virgibacillus halodenitrificans* DASH were determined; it exhibited the better performance for both growth and orange pigment production (364 μg/mL) under mesophilic conditions, pH 9 and availability of NaCl (2 M). Similarly, Asker et al. [45] and Xueqin et al. [46] found that higher pigment production was recorded by *Serratia marcescens* and *Virgibacillus halodentrificans* ST-1 under exact conditions. In the same sense, *Serratia* sp., *Micrcococcus* sp. and *Exiguobacterium* sp. PMA produced considerable pigments content at 30 °C [47,48,49]. On the other hand, *Bacillus* sp. showed significant pigment production under neutral conditions (pH 7.0 ± 0.1) and a temperature of 34 °C [50]. Interestingly, Hamidi et al. [51] listed the importance of NaCl in the growth of halophilic organisms. Moreover, Rodriguez-Valera et al. [52] found variability in pigment content upon altering salt concentrations in the growth medium of the genus *Halobacterium*. As revealed by Paliwal et al. [53], Al Disi et al. [54] and Allahkarami et al. [55], the nutrients are considered the most decisive factor in enhancing or suppressing the productivity of bioactive metabolites, and any alterations in nutrients composition and concentrations certainly influences the biochemical pathways that promote carotenogenesis process in microorganisms. Recent research efforts have focused on optimization and scaling up of environmental, incubation and nutritional factors to enhance the yield of microbial metabolites without increasing the process cost. Each organism has its own special conditions to maximize every particular product [24]. The main conventional strategy used in media engineering for obtaining the optimal operating conditions is one variable at time (OVAT). Such a single dimensional task does not explain the interaction effects among the variables on the production process. Moreover, it is a time consuming and laborious practice. To overcome these limitations, a statistical approach was applied [15].

Hence, PBD and RSM methods were employed in the current study to maximize the productivity of halophilic pigments. The statistical methods unveiled that yeast extract, peptone, NaCl and inoculum size were limiting parameters that governed the enhanced productivity of carotenoids. Likewise, El-Banna et al. [56] highlighted that complex organic sources such as soybean meal, soy peptone, beef extract and yeast extract generated higher carotenoids accumulation in *Rhodotorula glutinis*, as important macro- and micronutrients like amino acids, minerals and vitamins that support microbial cell growth are supplied by them. Also, Zhao et al. [57] mentioned the vital role of yeast extract in promoting carotenoid production by marine *Rhodotorula* sp. In contrast, Silva et al. [58] showed that varying the concentration of yeast extract from 1 g/L to 10 g/L did not show any positive effect on enhancing the carotenoid content of *Xanthophyllomyces dendrorhous*, reflecting strain specificity in their nutritional requirements. In the same sense, Allahkarami et al. [55] referred to the significance of glycerol in promoting carotenoids production by *R. glutinis* due to its better diffusion through the cell membrane and also inducing stress inside the cells, which stimulates the production of secondary metabolites. Interestingly, Hamidi et al. [51] confirmed the basic role of NaCl in promoting carotenoids content extracted from *Halorubrum* sp. 

Meanwhile, as secondary metabolites, pigments production was influenced by initial inoculum size. As referred to by Sumathi et al. [59], there is a positive correlation between initial inoculum concentration and fermentation byproducts, which resulted in accelerating the production process by reducing the generation time. Similarly, Abdel-Raheam et al. [60] found that the production of yellow, red and orange pigments by *Monascus ruber* was improved upon increasing the inoculum size to 81 × 10^4^ (spores/mL), elucidating that too little inoculum led to insufficient biomass and thereafter lower productivity of metabolites, while higher inoculum concentration generated excessive biomass which is considered an essential precursor for pigment formation, which harmonized with the results of the current study. Generally, the statistical methods of optimization succeeded in improving the carotenoids content of *V. halodenitrificans* DASH by 2.12-fold when compared to the basal medium.

Notably, the stability of extracted pigments under different processing is a significant parameter in industrial applications. As stated by Gutiérrez et al. [61] and Liang et al. [62], the employment of β-carotene as a food colorant is managed by solubility, stability, melting point and low bioavailability. However, β-carotene is quite unstable and degrades during food processing and storage [63]. Herein, the carotenoids extracted from *V. halodenitrificans* DASH exhibited considerable stability under dark, psychrophilic and neutral condition, and gradual decrease upon exposure to light, highly acidic/basic conditions and under temperature range of 25–50 °C. Similar studies were carried out by Shatila et al. [64] and showed that 80% stability in orange color pigment extracted from *Exiguobacterium aurantiacum* FH was obtained after exposure to light for 24 h. Likewise, orange pigment produced by *Kocuria* sp. BRI 36 displayed about 77% stability at 10 °C within 5 h, with a diminishing stability reaching 38% upon exposure to 50 °C for 5 h [65]. Broadly, to address and overcome the stability pitfall, immobilization by encapsulation could elevate the stability for subsequent applications in the food industry. In addition, to lessen any adverse effect of pigments’ methanolic extract in subsequent applications, even insignificant, the lyophilized formula of pigments was recommended, as the lyophilization process avoids using heat, which therefore ensures materials’ un-degradability and maintains their stability in room temperature and prolongs their shelf life.

Notably, several analytical techniques were used to characterize and determine the identity of orange pigment, including UV-Vis spectrum, FTIR, Raman spectroscopy, TLC and LC–MS. Regarding UV-Vis spectrum, Rajan and Gargi [66] detected the λmax at 490 nm for estimation of carotenoid content in pigment extracted from two yeast strains of *Rhodotorula mucilaginosa*. Additionally, Rajan and Gargi [66] detected such a discriminatory peak at the same wavelength when analyzing the pigments extracted from *R. mucilaginosa* (MTCC-1403), which both supported our finding. In general, the presence of a unique peak near to this region is highly characteristic for carotenoid compounds as pointed out by Vila et al. [33]. Moreover, based on LC–MS analysis data, it is safe to identify the orange pigment of *V. halodenitrificans* DASH as a mixture of β-carotene (*m*/*z* 536.8) (Carotene), lutein (*m*/*z* 568.8) (Hydroxycartenois) and β-Apo-8′-carotenal (oxocarotenoid) (*m*/*z* 416.6). Remarkably, Asker [67] found the same results when examining lutein and its isomer zeaxanthin produced by *Sphingobacterium multivorum*. Furthermore, the results of the current study agreed with what was reported by Etzbach et al. [68] concerning the identification of β-carotene produced by *S. senegalensis* fruits (*m*/*z* 536.1). 

Recently, the prevalence of MDR pathogens counts as a significant challenge; therefore, scientists and biotechnologists keep endeavoring to find alternative antimicrobial agents, especially from natural sources. Hence, carotenoids with their different types and isolation sources remain propitious due to their considerable effectiveness and lower toxicity impact on human health. Regardless of the difference in cell wall composition and susceptibility of each pathogen, the carotenoids of *V. halodenitrificans* DASH inhibited the growth of both Gram-positive and Gram-negative bacteria alike. Along the same lines, Manimala and Murugesan [69] mentioned the effectiveness of carotenoids extracted from *Sporobolomyces sp.* in inhibiting the growth of *E. coli* and *S. aureus* by 28 and 26 mm as ZOI. Additionally, ZOI of *Rhodococcus rhodochrous* pigment recorded 21 mm against *S. aureus* [70]. Notably, a robust mycocidal capability was observed against *C. albicans*, implying the disruption of the glycoprotein-glucan-chitin cross-linkage of fungi cell wall and malfunctioning in biochemical physiological processes. Seemingly, *V. halodenitrificans* DASH carotenoids could show a promising efficacy in frustration of COVID-19 post infections that are identified as white pathogenic fungi. Such fungal post infections emerged recently in the second wave in India and were followed by other countries, particularly in patients who suffered from abnormal breathing in intensive care units. Commonly, the exact antimicrobial strategies for carotenoids are still ambiguous; however, the higher penetration through the cells suppresses microbial growth via possible scenarios including periplasmic membrane permeability, cytoplasmic content leakage, protein inhibition, arresting nucleic acids functionalities and generation of reactive oxygen species (ROS) [71,72]. 

Furthermore, the antibiofilm potency of *V. halodenitrificans* DASH carotenoids, which increased linearly with increasing the applied dosage of carotenoid, denotes a dose dependent behavior of inhibition. In agreement with our results, Jemil et al. [73] proved that carotenoids extracted from haloarchaea possessed antibiofilm activity. The strategies followed by the carotenoids to prohibit biofilm formation are via diminishing cell adhesion, hindering EPS matrix formation and more significantly impeding the network of quorum sensing [72]. As revealed by Sampathkumar et al. [42], lutein extracted form *Chlorella pyrenoidosa* (20 μg/mL) showed noticeable inhibition against *P. aeruginosa* biofilm, pyocyanin production and degradation of extracellular polymeric substances. In view of the current results, the methanolic extract of *V. halodenitrificans* DASH carotenoids exhibited a promising potentiality versus different pathogens, either in free-living or biofilm lifestyles, which encourages their recruitment for defeating microbial threats in a variety of fields including environmental, agricultural, the food-processing industry, the cosmetics industry, and pharmaceutical and medicinal disciplines.

## 5. Conclusions

To summarize, the carotenoids extracted from halophilic *V. halodenitrificans* DASH that was screened from the El-Natrun salt lakes, were optimized using statistical means. On optimized medium, the halophilic carotenoids were characterized using UV-Vis spectrum, FTIR, Raman spectroscopy, TLC and LC–MS. UV-Vis spectrum detected a unique peak at 490, which distinguishes carotenoids. However, the existence of functional groups such as C-O-H, CH3, C-H, C=C, C-CH, -CH2-, C-O, C=O and –OH groups was confirmed via FTIR and Raman spectroscopy. Meanwhile, TLC analysis revealed two distinct orange and yellow bands at retention times of 0.82 and 0.67, respectively, whereas LC–MS identified their ingredients. Moreover, the storage experiments revealed that the tested carotenoids were stable under dark, psychrophilic and neutral conditions, with gradual decrease upon exposure to light, highly acidic/basic conditions and under temperature range 25–50 °C. Furthermore, upon application, the biological activity of the carotenoids was evaluated in terms of antimicrobial and antibiofilm activity. The results confirmed the antibacterial, fungicidal and antibiofilm properties in response to a wide spectrum of pathogens, which opens up the door to replace or promote traditional antimicrobial medications for beating different forms of MDR pathogens that represent serious environmental and medical issues.

## Figures and Tables

**Table 2:** ANOVA for quadratic model of pigment extracted from *V. halodenitrificans* DASH.

Source	DF	Seq SS	Adj SS	Adj MS	F	*p*
Main Effects	8	0.33246	0.33246	0.04156	19.1	0.017
Residual Error	3	0.00653	0.00653	0.00218		
Total	11	0.33899				

**Table 3:** Central composite design (CCD) representing matrix and pigment weight (mg/mL) extracted from *V. halodenitrificans* DASH as influenced by yeast extract, peptone, NaCl concentrations and inoculum size (%), along with the predicted pigment weight, residuals and concentrations of variables at each level.

Run Order	Yeast Extract (g)	Peptone (g)	NaCl (g)	Inoculum Size (%)	Experimental Pigment Weight (mg/mL)	Predicted Pigment Weight (mg/mL)	St. Residual
1	−1	1	−1	1	7.89	9.84	−1.24
2	0	0	0	2	18.57	20.78	−1.41
3	1	−1	−1	−1	12.31	13.21	−0.58
4	−1	1	1	−1	6.68	8.05	−0.88
5	2	0	0	0	8.36	10.21	−1.18
6	1	−1	1	−1	8.68	9.31	−0.39
7	1	1	1	−1	13.36	12.73	0.4
8	−1	−1	−1	1	15.73	14.57	0.75
9	0	0	0	0	22.36	21.1	0.57
10	1	−1	1	1	12.89	10.36	1.62
11	1	1	−1	−1	6.26	6.47	−0.15
12	−1	−1	1	1	7.0	9.31	−1.49
13	−1	−1	1	−1	0.263	1.47	−0.76
14	1	1	1	1	12.36	12.68	−0.19
15	0	0	0	0	21.1	21.1	0
16	0	0	0	0	21.68	21.1	0.26
17	−1	−1	−1	−1	3.05	5.31	−1.45
18	0	0	2	0	0.21	0.631	−0.26
19	1	1	−1	1	10.78	7.84	1.91
20	0	−2	0	0	17.36	15.36	1.28
21	1	−1	−1	1	14.52	15.68	−0.76
22	−1	1	−1	−1	1.0	1.73	−0.47
23	0	0	0	0	20.78	21.1	−0.14
24	0	0	−2	0	0.789	0.368	0.75
25	−2	0	0	0	6.94	4.36	1.66
26	0	0	0	0	20.94	21.1	−0.07
27	0	2	0	0	12.84	14.1	−0.8
28	0	0	0	0	17.73	21.1	−1.51
29	0	0	0	0	23.1	21.1	0.9
30	0	0	0	−2	14.52	11.57	1.89
31	−1	1	1	1	17.42	14.73	1.73
**Variable**			**Coded Levels/Experimental Values**				
			**−2**	**−1**	**0**	**1**	**2**
**Yeast Extract (g)**			0.5	1	2	4	7
**Peptone (g)**			3	5	10	15	20
**NaCl (g)**			58.4	116.8	233.6	350.4	467.2
**Inoculum Size (%)**			0.1%	0.3%	0.5%	2%	4%

## Data Availability

Data are contained within the article.

## Supplementary Materials

The following supporting information can be downloaded at: https://www.mdpi.com/article/10.3390/biology11101407/s1, Table S1. Statistical analysis of Plackett–Burman design showing coefficient, *t*-test values and *p*-values of variables influencing pigment production; Table S2. Estimated effect, regression coefficients and corresponding T and *p* values in addition to ANOVA analysis for the optimization of pigment production using CCD. Figure S1. Normal probability plot of variables (A); Pareto chart of independent factors affecting pigment production (B); and Normal probability plot of residuals (C); Figure S2. The normal probability plot of the residuals for pigment weight by *V. halodenitrificans* DASH determined by the second-order polynomial equation; Figure S3. (A) Response optimizer with desirability function for pigment weight with maximum goal; Figure S4. Biological activity of halophilic carotenoids. (A) Antimicrobial activity against different pathogens via well diffusion method and (B) Microtiter plate assay showing the antibiofilm capacity of haloalkaliphilic carotenoids extracted from halophilic *V. halodenitrificans* DASH. A, a, b and c demonstrated control (before treatment), 10 and 20 μg/mL of carotenoid treatments against *P. aeruginosa* biofilm, respectively. d, e, f showed control (before treatment), 10 and 20 μg/mL of carotenoid treatments against *S. aureus* biofilm, respectively.
