# Supplementary material for: Carotegenic Virgibacillus halodenitrificans from Wadi El-Natrun Salt Lakes: Isolation, Optimization, Characterization and Biological Activities of Carotenoids
Source: Biology (Basel). 2022 Sep 27;11(10):1407. doi: 10.3390/biology11101407 (PMC9598633; doi:10.3390/biology11101407)

Carotegenic *Virgibacillus halodenitrificans* from Wadi El-Natrun salt lakes: Isolation, optimization, characterization and biological activities of carotenoids

Doaa Fayez[1], Asmaa Youssif[1], Soraya Sabry[1], Hanan Ghozlan[1*], and Marwa Eltarahony[2*]

[1] Botany and Microbiology Department, Faculty of Science, Alexandria University, 21321, Alexandria, Egypt.

[2] Environmental Biotechnology Department, Genetic Engineering and Biotechnology Research Institute (GEBRI), City of Scientific Research and Technological Applications (SRTA-City), 21934, New Borg El-Arab City, Alexandria, Egypt

* Corresponding authors: meltarahony@srtacity.sci.eg, m_eltarahony@yahoo.com (M.E); Tel.: +20-3459-3422; Fax: +20-3459-3407; ghozlan.hanan@alexu.edu.eg, hananghozlan@gmail.com (H.G)

Supplementary Table S1. Statistical analysis of Plackett–Burman design showing coefficient, *t*-test values and *P*-values of variables influencing pigment production.

Term	Effect	Coef	SE Coef	T	P
Constant		0.21242	0.0135	15.77	0.001
KCL	-0.003	-0.00158	0.0135	-0.12	0.914
$MgSO_4$	-0.029	-0.01442	0.0135	-1.07	0.363
Y. Extract	-0.111	-0.05558	0.0135	-4.13	0.026
Peptone	0.1408	0.07042	0.0135	5.23	0.014
NaCL	0.2595	0.12975	0.0135	9.63	0.002
Size	-0.096	-0.04808	0.0135	-3.57	0.038
PH	0.0055	0.00275	0.0135	0.2	0.851
V/flask	-0.034	-0.01708	0.0135	-1.27	0.294
**R^2** = 98.07%			**R^2 (adj)** = 92.94%		

Supplementary Table S2. Estimated effect, regression coefficients and corresponding *T* and *P* values in addition to ANOVA analysis for the optimization of pigment production using CCD

**Term**	**Coef**		**SE Coef**		**T**	***P***
**Constant**	0.401		0.017279		23.207	0
**Yeast Extract**	0.027708		0.009332		2.969	0.009
**Peptone**	-0.006125		0.009332		-0.656	0.521
**NaCL**	0.004708		0.009332		0.505	0.621
**Inoculum Size**	0.043625		0.009332		4.675	0
**(Yeast Extract)^2**	-0.065656		0.008549		-7.68	0
**(Peptone)^2**	-0.030281		0.008549		-3.542	0.003
**(NaCL)^2**	-0.099656		0.008549		-11.657	0
**(Inoculum Size)^2**	-0.023406		0.008549		-2.738	0.015
**Yeast Extract * Peptone**	-0.014938		0.011429		-1.307	0.21
**Yeast Extract * NaCL**	-0.000313		0.011429		-0.027	0.979
**Yeast Extract * Inoculum Size**	-0.032187		0.011429		-2.816	0.012
**Peptone * NaCL**	0.048312		0.011429		4.227	0.001
**Peptone * Inoculum Size**	-0.005562		0.011429		-0.487	0.633
**NaCL * Inoculum Size**	-0.006687		0.011429		-0.585	0.567
**Source**	**Df**	**Seq SS**	**Adj SS**	**Adj MS**	***F***	***P***
**Regression**	14	0.5031	0.5031	0.0359	17.19	0
**Linear**	4	0.0655	0.0655	0.0164	7.84	0.001
**Square**	4	0.3789	0.3789	0.0947	45.32	0
**Interaction**	6	0.0587	0.0587	0.0098	4.68	0.006
**Residual Error**	16	0.0334	0.0334	0.0021		
**Lack of fit**	10	0.0272	0.0272	0.0027	2.59	0.128
**Pure error**	6	0.0063	0.0063	0.001		
**Total**	30	0.5365				

Supplementary Figure S1. Normal probability plot of variables (A); Pareto chart of independent factors affecting on pigment production (B) and Normal probability plot of residuals (C)

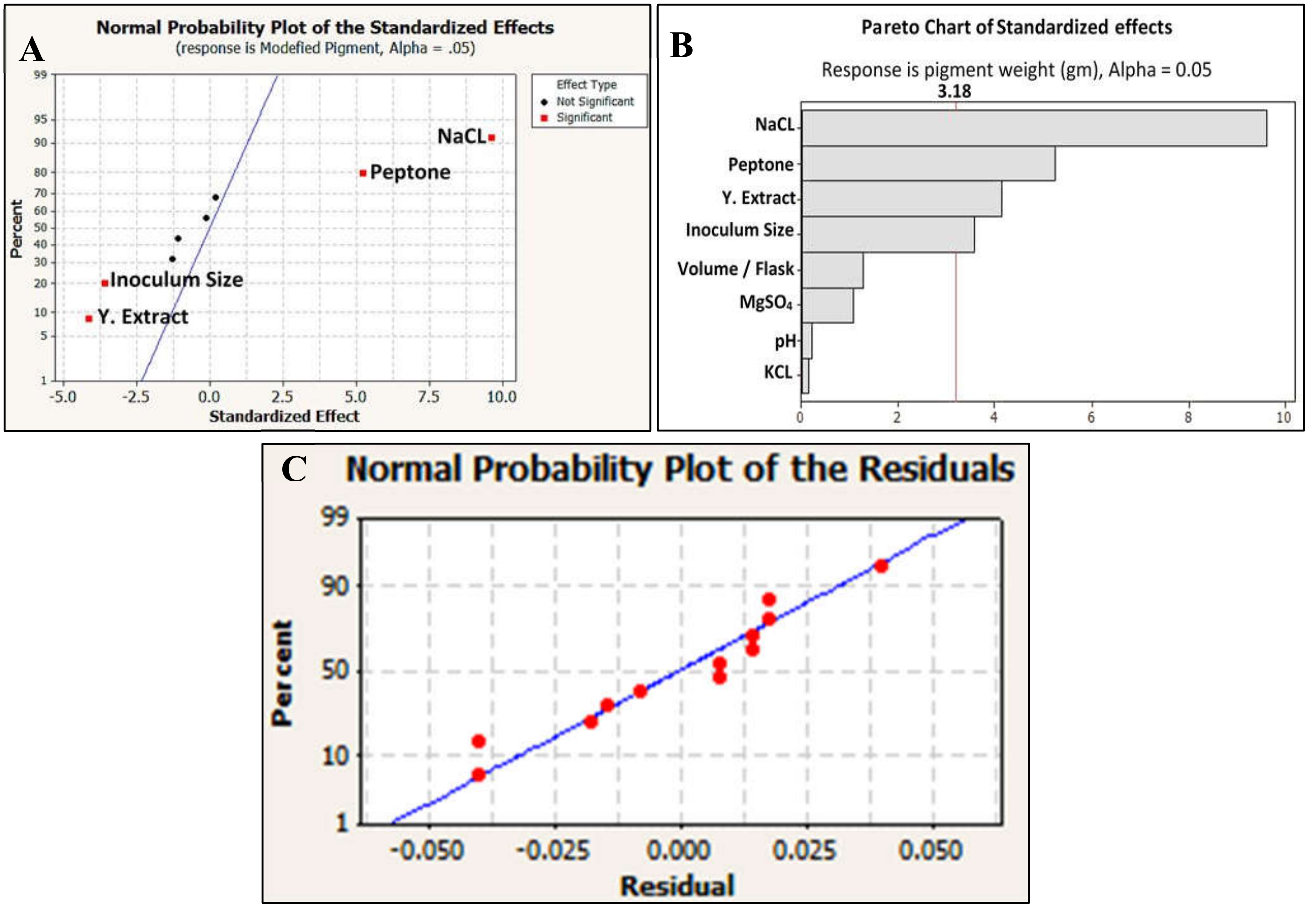

Basically, the probability plot of effects separates random noise from real effects based on their distribution on the plot. Where, the variables that lie furthest from the line consider being the most significant. However, the factors with negligible effect lie along the line. Accordingly, NaCL and peptone positively influenced pigment productivity as they lied on the right-hand side of the line (i.e. higher concentration of both ingredients was accompanied by higher yield of pigment). While, yeast extract and inoculum size influenced negatively as seen as lying on the left-hand side of the line (Supplementary Fig. S2-A), where, higher yield of pigment generated with lower values of both parameters. Besides, Pareto chart (Supplementary Fig. S2-B) pointed out to the order of significance of each independent parameter. It is clear from (Supplementary Fig. S2-C) that the residuals followed normal distribution as well as the majority of the data points are distributed along the diagonal line of pigment weight.

Supplementary Figure S2. The normal probability plot of the residuals for pigment weight by *V. halodenitrificans* DASH determined by the second-order polynomial equation

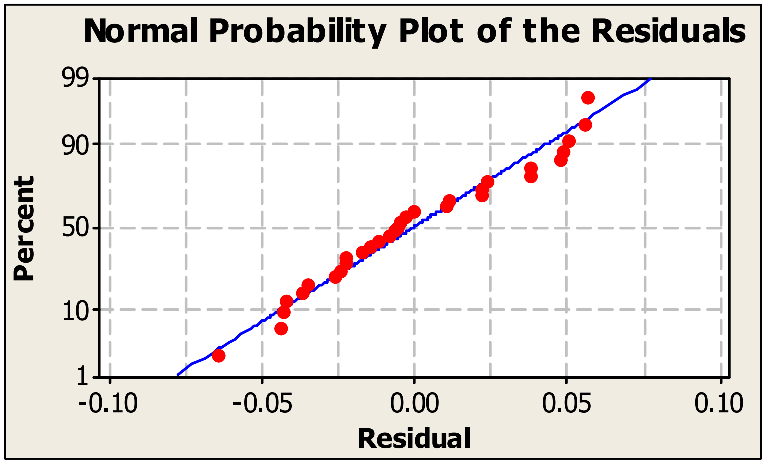

Supplementary Figure S3. Response optimizer with desirability function for pigment weight with maximum goal

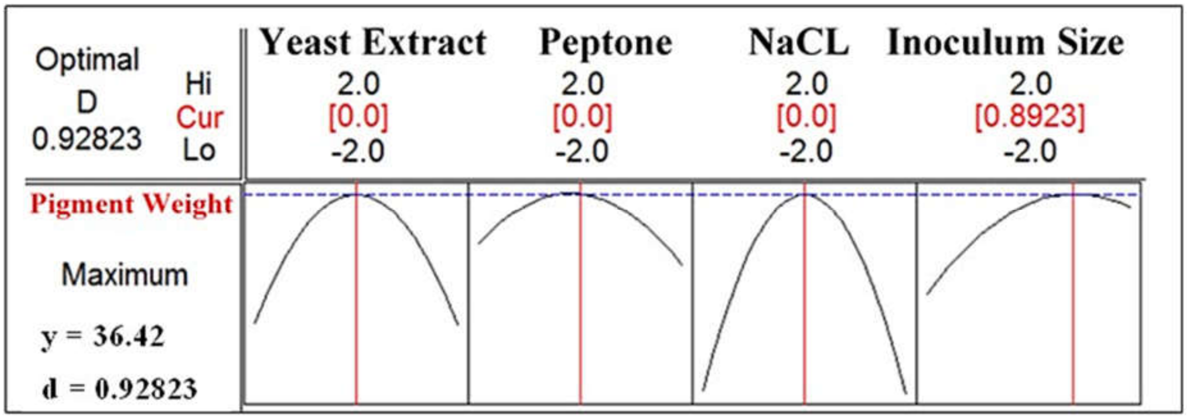

Supplementary Figure S4. Biological activity of halophilic carotenoids. (A)- Antimicrobial activity against different pathogens via well diffusion methode and (B)- Microtiter plate assay showing the antibiofilm capacity of haloalkaliphilic carotenoids extracted from halophilic *V. halodenitrificans* DASH. A, B and C demonstrated control (before treatment), 10 and 20 µg/mL of carotenoid treatments against *P. aeruginosa* biofilm, respectively. D, E, F showed control (before treatment), 10 and 20 µg/mL of carotenoid treatments against *S. aureus* biofilm, respectively.

A

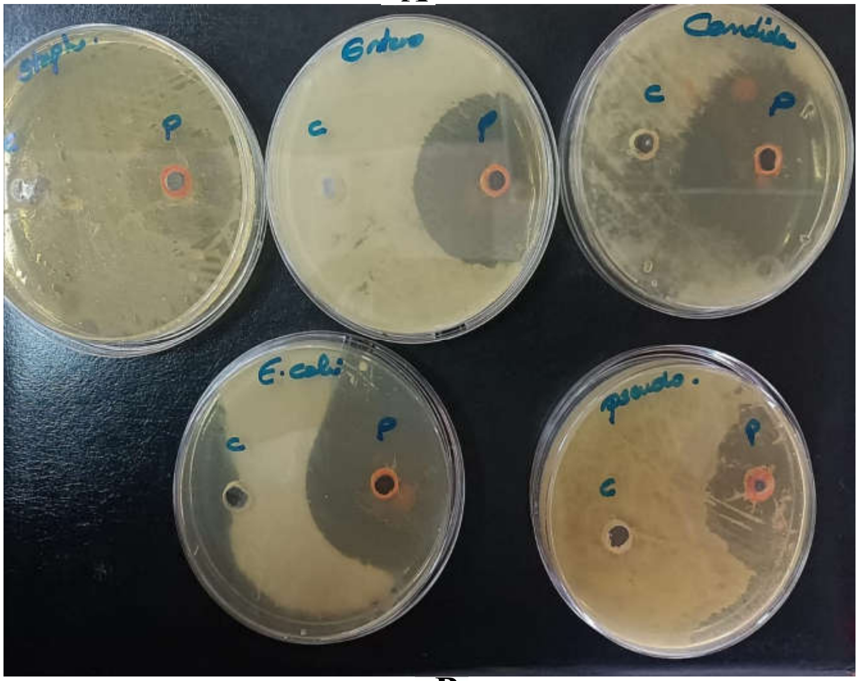

B

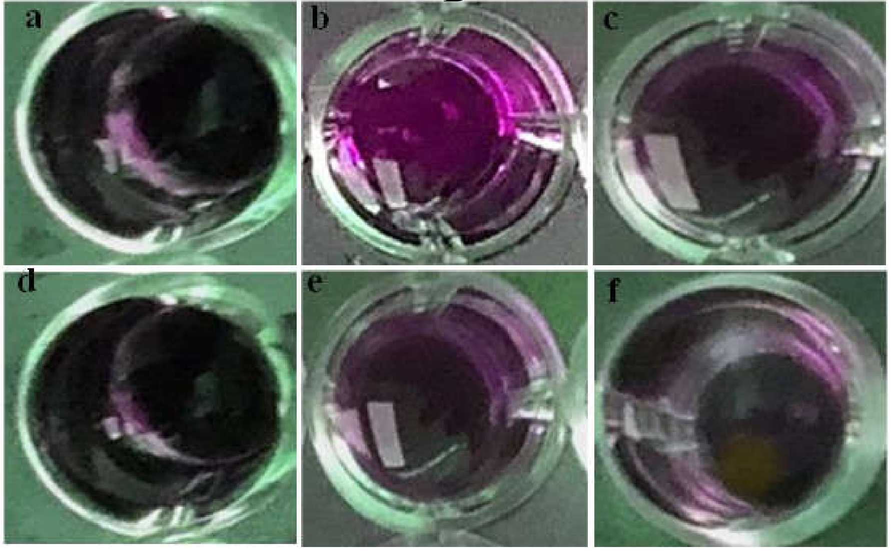

Supplement: Supplementary file 1 [file biology-11-01407-s001.zip › biology-1895599-supplementary.pdf]
